# Climate change, migration, and health(care) in primary care training

**DOI:** 10.4102/phcfm.v13i1.3227

**Published:** 2021-10-19

**Authors:** Charlotte Scheerens, Farai D. Madzimbamuto

**Affiliations:** 1 Faculty of Economics and Business Administration, Ghent University, Ghent, Belgium; 2 Department of Public Health and Primary Care, Faculty of Medicine and Health Sciences, Ghent University, Ghent, Belgium; 3 The Institute for Lung Health, Harvard Medical School, Boston, MA, United States of America; 4 Department of Anaesthetics and Critical Care, Faculty of Medicine, University of Botswsana, Gaborone, Botswana

Family physicians (FPs) are community-orientated primary care generalists. However, they are often underrepresented in research on climate change, migration and health (care).^1^ Research study shows that the interaction between these phenomena is complex and unfamiliar to many FPs in sub-Saharan Africa (SSA).^2^ Communities in SSA are already witnessing the consequences of climate change, such as extreme weather events. Migration, whether related to climate change, is often a health-seeking strategy, but may result in more health risks.^3^ There is an urgent need to raise public awareness about these interactions, and for primary care professionals (PCPs) to lead and advocate for climate-resilient, migration-inclusive health systems.^4^ Accelerated efforts to educate health professionals on the health effects of climate change through inclusion in training curricula and continuing education are needed.^2,5^

The following topics could be mainstreamed throughout training curricula for FPs and PCPs in Africa:

- interprofessional emergency planning, coordination and rapid response to adverse weather events and raised temperatures
- the impact of migration and forced displacement on urban overcrowding, poor water, sanitation and hygiene (WASH) infrastructure, communicable disease outbreaks, mental health and substance abuse, sexual and reproductive health and gender-based violence
- caring for both the host community’s and migrant’s health
- the impact of domestic and occupational environmental pollution and degradation (air, water, waste disposal, informal mining and agriculture)
- the impact of climate change on agriculture, food security and nutrition.

These topics should be taught using an approach that acknowledges the interactions and complex feedback loops connecting them. These are key study areas for planetary health, public health, environmental and occupational health, and emergency medicine, and speak to the need for greater interprofessional and multidisciplinary education and collaboration. The role of social determinants of health and equitable access to healthcare are the foundation of public health practice and community-oriented primary care (COPC), and should also form an integral part of family medicine training. Online educational packages based on systems thinking, which prepare participants to anticipate, mitigate, plan for, adapt and respond to the health impacts related to migration and climate change, are a critical way forward. The recently developed planetary health education framework^6^ and the free online World Organization of Family Doctors (WONCA)-environment course on planetary health for primary care^7^ are important guidance tools to integrate systems thinking and a planetary health approach in such packages.

Furthermore, enhancing research capacity through FP training in African countries is essential so that locally appropriate health interventions can be developed and evaluated, stories and narratives are documented, and evidence is generated on the health implications of migration and climate change. Closer collaboration with public health, environmental and occupational health specialists and researchers will facilitate research on disease surveillance in populations, disease patterns in relation to climate change, changes in service utilisation, and methods of strengthening health systems. Family physicians are in a unique position to contribute case studies on building resilience in communities and influencing priority setting for research, as well as guiding opinion-leaders and policymakers.

Alongside the proposed training reform and research capacity strengthening, broader international support is needed to provide sustainable solutions for African communities at risk of climate change and migration. African countries are now bearing the brunt of climate change despite contributing the least to the underlying causes. Expectations that African countries will forego economic development based on coal, oil and gas, which funds national education and health plans, are only realistic if balanced with considerable investment in renewable energy infrastructure. Similarly, massive investment in urban and peri-urban WASH facilities will be necessary to avoid repeated outbreaks of communicable diseases. High-income countries should compensate affected countries, support their development and be held more accountable, especially in how development aid is used to benefit their populations’ livelihoods. Research and training support could be provided through academic partnerships with African universities, including local sponsorship and scholarships. The International Thematic Network CliMigHealth (a Primafamed and University of Ghent partnership launched in 2020), for example, facilitates research and education partnership in Africa concerning the climate change, migration and health nexus. The team is currently developing an education package on the nexus for FPs and PCPs with Stellenbosch University, who will pilot test it in their postgraduate FP training.^8^

Family physicians and PCPs can provide valuable insights into multisectoral action on health, education, social services, agriculture, food security and poverty alleviation for climate-resilient and migration-inclusive health systems. They are trusted by local communities and regarded as role models for their patients who advocate on their behalf to national governments, industry leaders, private and civil society organisations, to uphold their responsibilities and obligations.^2^ Communication and advocacy skills should thus be implemented in FP training, so that FPs are better prepared to take up these roles in the community. The World Health Organization (WHO) Operational Framework for climate-resilient health systems, with its integrated development strategies and governance for local, national and international governments and agencies, can be used as an advocacy tool in training but with more emphasis on communications and advocacy and the connection with migration and related health risks should also be made more explicit.^9^ Such actions will strengthen the overall health service delivery, as emphasised in the Astana Declaration on primary health care (PHC) and the Global Sustainable Development Goals.
